# Klotho suppresses the inflammatory responses and ameliorates cardiac dysfunction in aging endotoxemic mice

**DOI:** 10.18632/oncotarget.14933

**Published:** 2017-02-01

**Authors:** Haipeng Hui, Yufeng Zhai, Lihua Ao, Joseph C. Cleveland, Hongbin Liu, David A. Fullerton, Xianzhong Meng

**Affiliations:** ^1^ Department of Surgery, University of Colorado Denver, Aurora, USA; ^2^ Department of Geriatric Cardiology, PLA General Hospital, Beijing, China

**Keywords:** aging, endotoxemic cardiac dysfunction, inflammation, Klotho, heat shock protein

## Abstract

**Background:**

Aging augments endotoxemic cardiac dysfunction, but the mechanism remains unclear. Anti-aging protein Klotho has been found to modulate tissue inflammatory responses. We tested the hypothesis that a reduced Klotho level in aging heart plays a role in the augmented endotoxemic cardiac dysfunction.

**Materials and Methods:**

Endotoxin (0.5 mg/kg, iv) was injected to adults (4-6 months) and aging (18-20 months) C57BL/6 mice. Recombinant Klotho (10 μg/kg, iv) was administered to a group of aging mice after endotoxin injection. Cardiac function was analyzed using a microcatheter at 24 and 48 h after endotoxin administration. Myocardial levels of Klotho and heat shock protein 70 (HSP70) were determined by immunoblotting, and plasma and myocardial cytokines were analyzed using ELISA.

**Results:**

More severe cardiac dysfunction in aging mice were accompanied by greater cytokine levels in the plasma and myocardium. Klotho was detected in the myocardial tissue. Klotho levels were lower in aging hearts and were further reduced during endotoxemia. Myocardial HSP70 levels were correlated with Klotho levels. Recombinant Klotho increased myocardial HSP70, inhibited NF-κB activation, reduced cytokine levels, and improved cardiac function in aging endotoxemic mice. Delivery of HSP70 into cultured macrophages suppressed endotoxin-induced NF-κB activation.

**Conclusions:**

Aging-related augmentation of inflammatory responses and cardiac dysfunction is associated with relative Klotho deficiency. Post-treatment with recombinant Klotho suppresses the inflammatory responses and improves cardiac function in aging endotoxemic mice. Klotho modulates HSP70 levels and HSP70 appears to be involved in the anti-inflammatory mechanism of Klotho. Klotho may have therapeutic potential in amelioration of aging-related endotoxemic cardiac dysfunction.

## INTRODUCTION

Trauma and stress associated with major surgery can result in endotoxemia and the systemic inflammatory response by eliciting gut bacteria translocation [1]. It is well known that endotoxemia causes cardiovascular dysfunction. While the general immunological function is decreased with aging, the inflammatory response to endotoxin (lipopolysaccharide, LPS) is enhanced in old animals [2–5]. Toll-like receptor 4 (TLR4) plays a central role in the regulation of LPS signaling and LPS-induced production of multiple pro-inflammatory mediators [6]. Up-regulated production of pro-inflammatory cytokines in the myocardium, such as tumor necrosis factor-α (TNF-α) and interleukin-1β (IL-1β) plays an important role in cardiac contractile depression during endotoxemia [7–10]. We previously observed that the augmented inflammatory responses in aging endotoxemic mice, in comparison to adult endotoxemic mice, leads to more severe cardiac dysfunction [5]. Currently, the mechanism by which aging augments the inflammatory responses to endotoxemia is incompletely understood. As major surgeries performed in the elderly is increasing, understanding of the impact of endotoxemia and inflammatory responses on cardiac function in aging subjects is helpful for improving post-surgery outcomes in the elderly.

Klotho is an anti-aging protein that is identified initially in the kidney [11]. Now, expression of Klotho has been found in several other tissues, including blood vessels [12]. Klotho has been found to regulate energy metabolism, exert anti-inflammatory and anti-oxidative effects, and modulate calcium and mineral homeostasis [13–15]. Klothos has also been implicated in vascular health and disease [16], and several studies suggest that Klotho has a cardioprotective effect [17]. Further, Klotho appears to modulate tissue inflammatory responses to injury or interact with inflammatory mediators [18, 19]. However, the mechanism by which Klotho exerts a cardioprotective effect remains to be determined. Further, it is unclear whether Klotho is expressed in the heart and whether aging and endotoxemia have an impact on myocardial Klotho levels.

Heat shock protein 70 (HSP70) is an inducible isoform of the 70 KD heat shock protein family and protects cells and tissue against stress and injury [20]. HSP70 can modulate the response to inflammatory cytokines, such as TNF-α and IL-1β [21]. Additionally, HSP70 could modulate the inflammatory status, preventing the inflammatory tissue damage caused by the aging-related chronic inflammation [22, 23]. Currently, the impact of aging on myocardial HSP70 levels is unclear.

In the present study, we tested the hypotheses that the worse cardiac dysfunction in aging endotoxemic mice is associated with reduced levels of Klotho in the myocardium and that HSP70 is involved in the anti-inflammatory mechanism of Klotho. We further proposed that recombinant Klotho has the therapeutic potential in mitigation of the inflammatory responses and cardiac dysfunction in aging endotoxemic mice. The present study determined: (1) the basal levels of Klotho and HSP70 in aging mouse heart and the effect of endotoxemia on their levels, (2) whether augmented inflammatory responses and more severe cardiac dysfunction in aging endotoxemic mice are associated with relative Klotho deficiency, (3) the relationship between Klotho and HSP70 during endotoxemia in aging mice and (4) the therapeutic potential of recombinant Klotho for amelioration of aging-related inflammation and cardiac dysfunction.

## RESULTS

### Endotoxemia causes more severe and sustained LV dysfunction in aging mice

**Figure 1:**
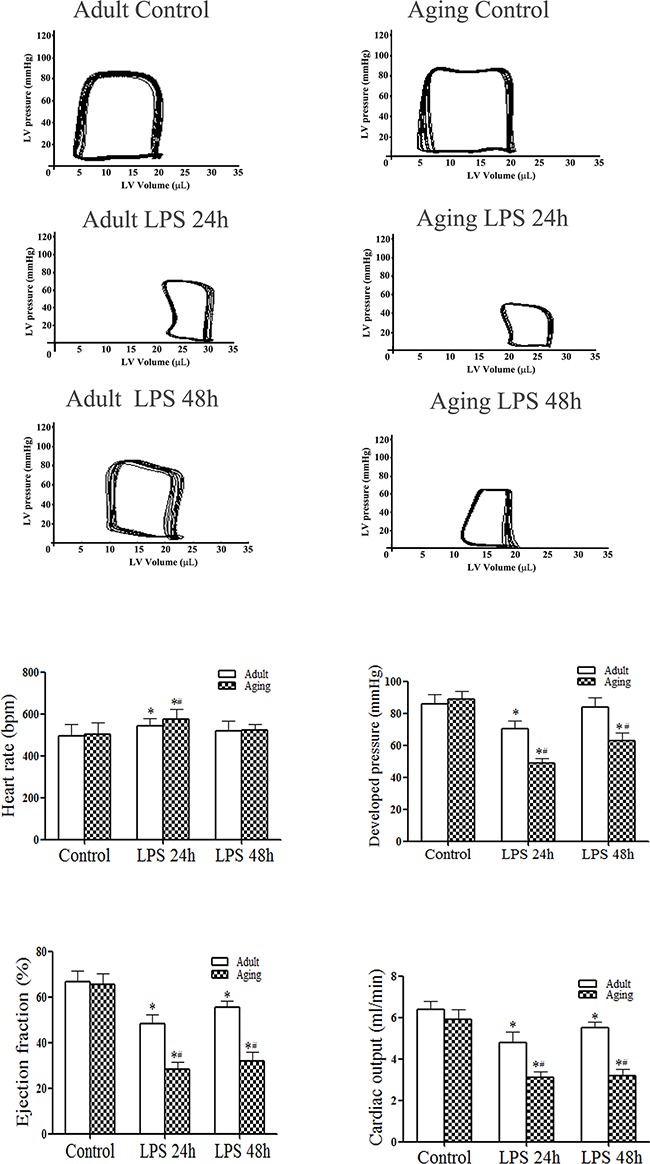
Endotoxemia results in greater and sustained left ventricular functional injury in aging mice Adult and aging mice were treated with lipopolysaccharide (LPS, 0.5 mg/kg, iv) or normal saline. Left ventricular (LV) pressure-volume loops were analyzed at 24 and 48 h after treatment. **A**. Representative pressure-volume loops show that aging mice display a greater reduction in LV performance. **B**. LV function data show that aging mice have lower developed pressure, ejection fraction and cardiac output after treatment with LPS. Data are expressed as mean ± SE. n=8, *P<0.05 vs. control, ^#^*P*<0.05 vs. the same time point in adult.

We previously reported that at 6 h after admini-stration of LPS, aging mice display greater cardiac depression in comparison to adult mice [5]. In the present study, we prolonged the time course. The pressure-volume loops presented in Figure 1A show representative LV function in terms of temporal pressure and volume. Compared to the saline control group, developed pressure, ejection fraction and cardiac output were lower at 24 h in LPS-treated adult or aging mice. However, significantly greater decreases in LV function parameters were observed in aging mice (Figures 1A and 1B). At 48 h after LPS administration, developed pressure was recovered to the baseline in adult mice, but remained lower than the baseline in aging mice. Although ejection fraction and cardiac output were improved in adult mice at this time point, they were comparable to those at 24 h in aging mice (Figures 1A and 1B). Thus, endotoxemia causes more severe and sustained LV dysfunction in aging mice.

Administration of LPS increased chemokine (MCP-1 and KC) and cytokine (TNF-α, IL-1β and IL-6) levels in the myocardial tissue and plasma in both adult and aging mice at 24 and 48 h (Tables 1 and 2). In comparison to adult mice, aging mice had greater levels of chemokine and cytokine in the myocardium and plasma at both time points (Tables 1 and 2). Therefore, the more severe and sustained LV dysfunction in aging mice was associated with augmented inflammatory responses.

**Table 1:** Myocardial chemokine and cytokine levels are higher in aging endotoxemic mice

Cytokines	Adult			Aging		
	Control	LPS 24 h	LPS 48 h	Control	LPS 24 h	LPS 48 h
MCP-1 (ng/mg)	80.8±16	328±31*	150±24*	104±21	804±88*^#^	399±78*^#^
KC (ng/mg)	81.6±24	501±79*	286±69*	93.8±17	1205±36*^#^	743±41*^#^
IL-6 (ng/mg)	71.4±9.5	264±18*	151±14*	83.8±5.4	493±30*^#^	345±21*^#^
IL-1β (pg/mg)	78.1±10	292±50*	152±35*	88.4±15	668±61*^#^	397±55*^#^
TNF-α (pg/mg)	15.3±5.1	160±32*	77±23*	19.3±4.4	387±43*^#^	278±29*^#^

Data are expressed as mean ± SE. n=8 in each of the 6 groups; *P<0.05 vs. control; ^#^*P*<0.05 vs. the same time point in adult

**Table 2:** Chemokine and cytokine levels in plasma are higher in aging endotoxemic mice

Cytokines	Adult			Aging		
	Control	LPS 24 h	LPS 48 h	Control	LPS 24 h	LPS 48 h
MCP-1 (ng/ml)	95.3±11.0	452±80*	247±21*	120±30.1	1201±66.3*^#^	433±87.7*^#^
KC (ng/ml)	58.1±17.8	248±35*	167±15*	57.1±19.8	472±39.5*^#^	277±49.1*^#^
IL-6 (ng/ml)	33.5±14.6	947±158*	198±47*	39.6±13.2	2746±307*^#^	1034±213*^#^
IL-1β (pg/ml)	45.5±7.0	435±49*	229±23*	50.8±10.0	1088±100*^#^	407±96*^#^
TNF-α (pg/ml)	35.8±17.2	521±113*	125±28*	33.1±14.3	1135±122*^#^	425±86*^#^

Data are expressed as mean ± SE. n=8 in each of the 6 groups; *P<0.05 vs. control; ^#^*P*<0.05 vs. the same time point in adult

### Endotoxemia reduces Klotho and HSP70 protein levels in hearts of aging mice

**Figure 2:**
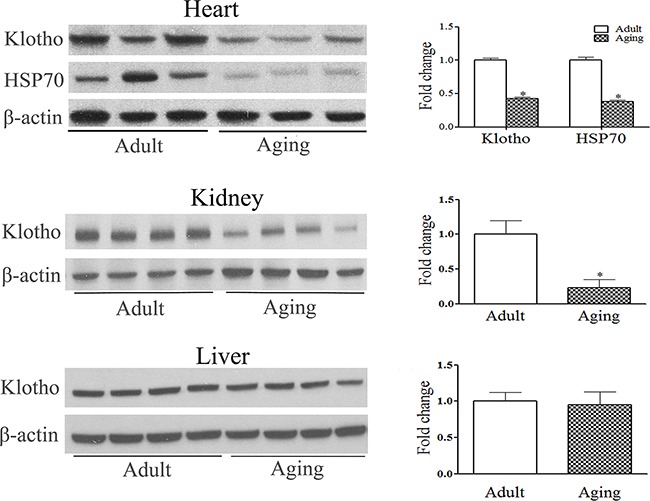
Relative Klotho deficiency in aging hearts is associated with lower levels of HSP70 Baseline levels of Klotho in the heart, kidney and liver were analyzed with immunoblotting and densitometric analysis. Lower levels of Klotho are evident in the heart and kidney of aging mice, but not in the liver. Relative Klotho deficiency in aging hearts is associated with lower levels of myocardial HSP70. Data are expressed as mean ± SE. n=6, *P<0.05 vs. adult.

Whereas Klotho levels were lower in the kidneys of aging mice, no age-related difference in liver Klotho levels was observed (Figure 2). Klotho was detectable in the myocardial tissue, and its levels in aging hearts were markedly lower compared to those in adult hearts (Figure 2). Aging hearts also displayed lower levels of HSP70 (Figure 2). Thus, Klotho protein is present in the heart, and reduced levels of myocardial Klotho in aging mice is accompanied by lower levels HSP70 in the myocardium.

**Figure 3:**
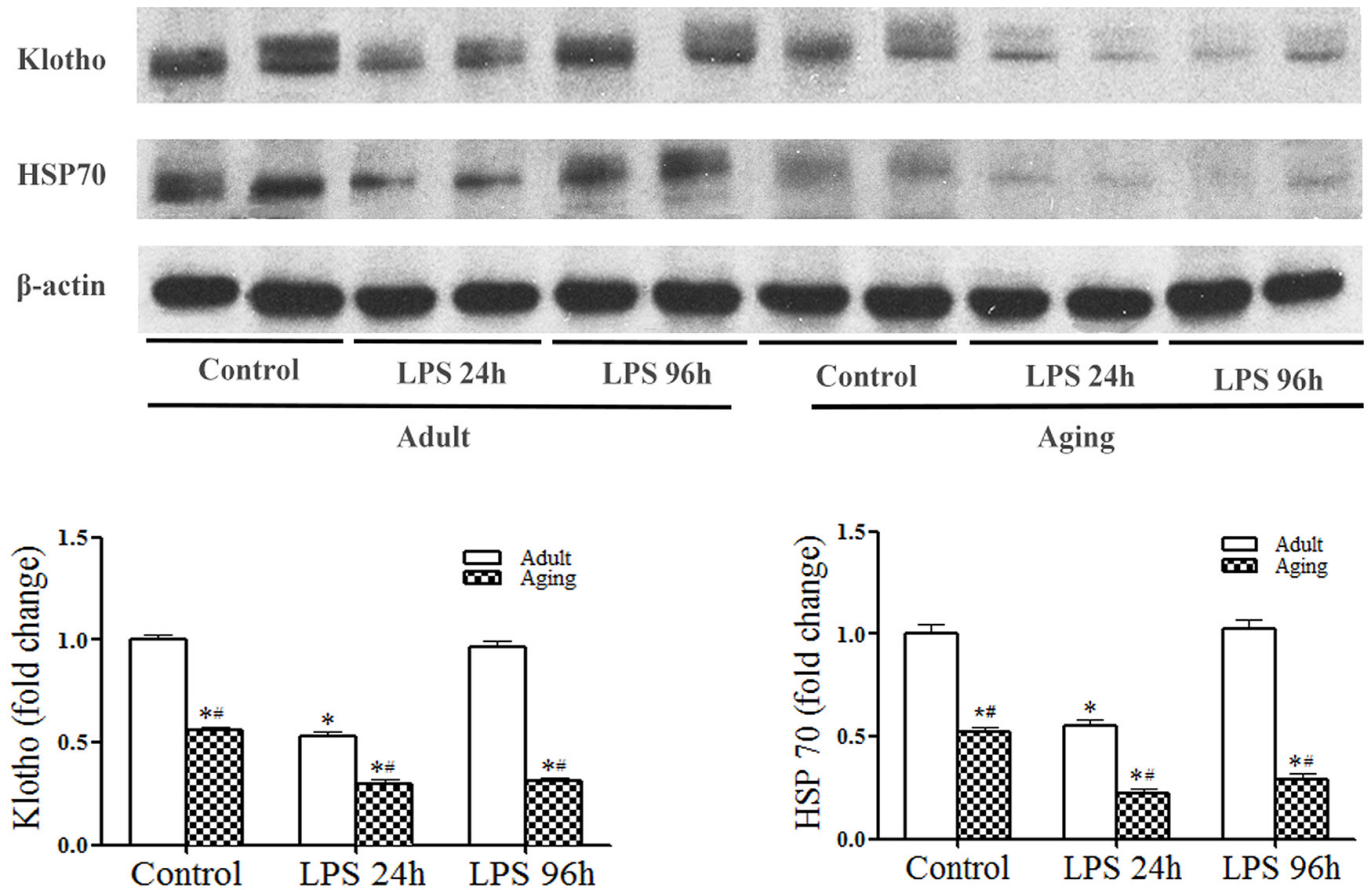
Endotoxemia causes a further reduction of Klotho and HSP70 levels in aging mice Adult and aging mice were treated with lipopolysaccharide (LPS, 0.5 mg/kg, iv) or normal saline. The levels of Klotho and HSP70 in myocardial tissue were analyzed with immunoblotting at 24 and 96 h after treatment. Representative immunoblots and densitometric data show that endotoxemia causes a reduction in myocardial Klotho and HSP70 levels at 24 h in adult mice, and myocardial levels Klotho and HSP70 are recovered to the baseline at 96 h. In aging mice, Klotho and HSP70 levels are also reduced at 24 h, but remain low at 96 h following LPS treatment. Data are expressed as mean ± SE. n=5, *P<0.05 vs. control, ^#^*P*<0.05 vs. adult.

Interestingly, we observed that endotoxemia caused a further reduction of cardiac Klotho and HSP70 levels (Figure 3). In adult hearts, Klotho and HSP70 levels were reduced at 24 h and recovered at 96 h after injection of LPS. However, Klotho and HSP70 levels were much lower at 24 h in aging hearts and failed to recover by 96 h (Figure 3). It appears that endotoxemia reduces myocardial levels of Klotho and HSP70, and it has a greater impact on aging hearts.

### Recombinant Klotho preserves myocardial HSP70 levels and improves LV function

**Figure 4:**
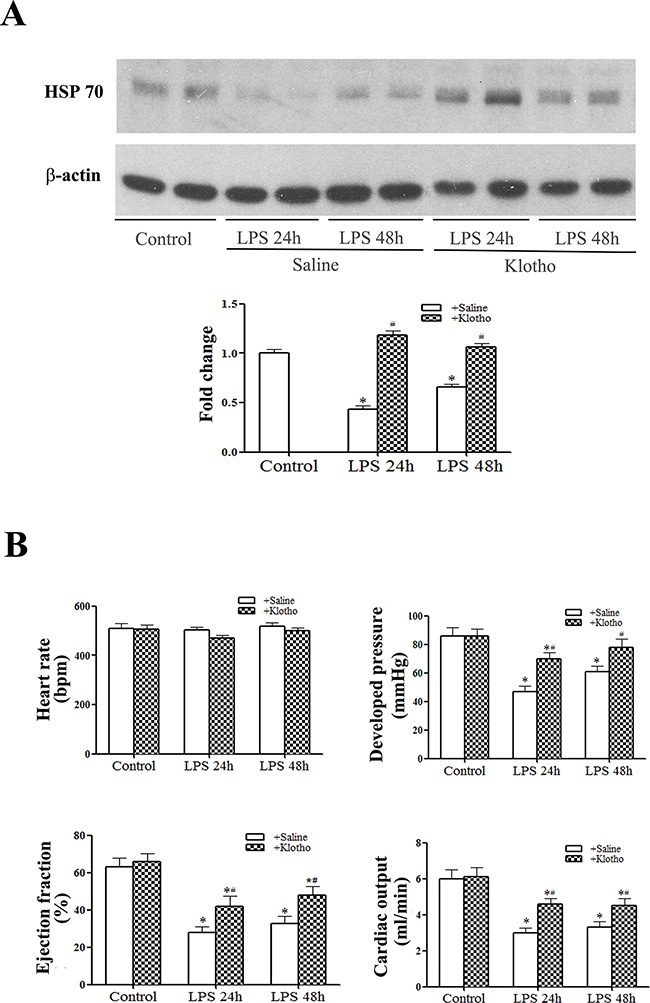
Treatment with recombinant Klotho after injection of LPS preserves myocardial HSP70 levels and improves cardiac function in aging mice Recombinant Klotho (10 μg/kg, iv) was administered 30 min after injection of lipopolysaccharide (LPS, 0.5 mg/kg, iv) in aging mice. Myocardial HSP70 levels were analyzed with immunoblotting, and LV pressure-volume loops were analyzed at 24 and 48 h after treatment with LPS. **A**. Representative immunoblot and densitometric data show that HSP70 levels are higher in aging mice treated with recombinant Klotho than those in aging mice treated with normal saline. **B**. LV function indices in aging mice are improved by the treatment with recombinant Klotho. Data are expressed as mean ± SE. n=6, *P<0.05 vs. control, ^#^*P*<0.05 vs. LPS+saline.

We used recombinant Klotho to determine the effect of Klotho on myocardial HSP70 levels and cardiac dysfunction in aging mice. Aging mice treated with recombinant Klotho after administration of LPS had higher HSP70 levels in the myocardium than aging mice treated with LPS+saline (Figure 4A). More importantly, cardiac function was markedly improved in aging endotoxemic mice treated with recombinant Klotho (Figure 4B). These results indicate that relative Klotho deficiency plays a role in myocardial HSP70 decline and cardiac dysfunction in aging endotoxemic mice.

### Klotho suppresses myocardial NF-κB activation and inflammatory mediator production

To determine the effect of recombinant Klotho on myocardial inflammatory responses in aging endotoxemic mice, we analyzed myocardial NF-κB activation and inflammatory mediator production.

**Figure 5:**
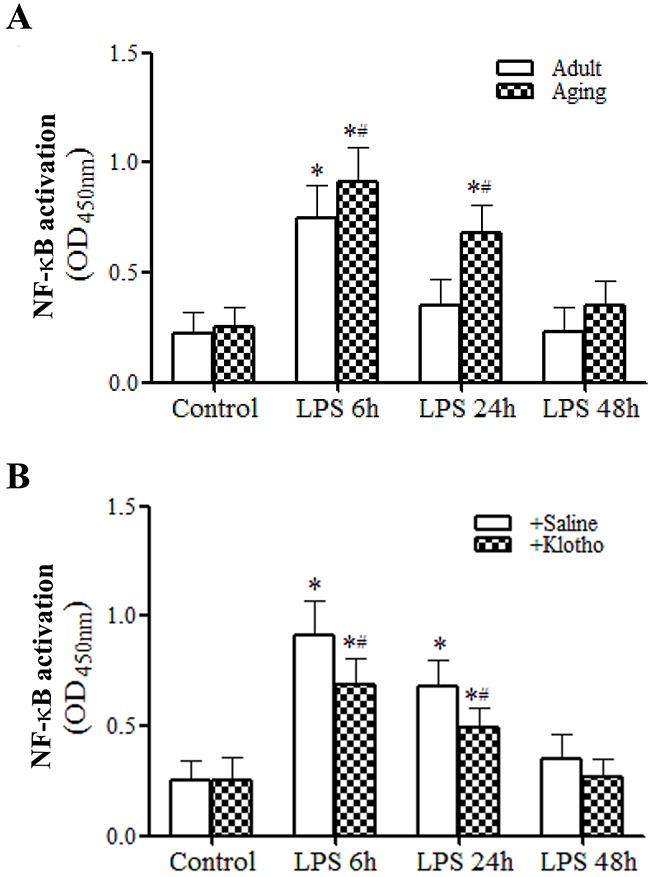
Recombinant Klotho suppresses myocardial NF-κB activation in aging endotoxemic mice **A**. Myocardial NF-κB activation was assessed using TransAM NF-κB activity assay kit. Myocardial NF-κB activity is higher and NF-κB activation lasts longer in aging endotoxemic mice. Data are expressed as mean ± SE. n=8, *P<0.05 vs. control, ^#^*P*<0.05 vs. adult. **B**. Treatment with recombinant Klotho after injection of LPS suppresses myocardial NF-κB activation. Data are expressed as mean ± SE. n=6, *P<0.05 vs. control, ^#^*P*<0.05 vs. LPS+saline.

As shown in Figure 5A, myocardial NF-κB activity was elevated at 6 h after LPS treatment and returned to the baseline level in adult mice. In contrast, myocardial NF-κB activity was higher at 6 h and remained elevated at 24 h in aging mice (Figure 5A). Thus, a sustained myocardial NF-κB activation in aging hearts was associated higher myocardial levels of chemokines and cytokines observed at 24 and 48 h (Table 1).

Treatment of aging mice with recombinant Klotho after administration of LPS decreased the levels of myocardial NF-κB activation at both 6 and 24 h (Figure 5B). Similarly, treatment of aging mice with recombinant Klotho after administration of LPS markedly reduced the inflammatory responses, lowering chemokine and cytokine levels in the myocardium and plasma at 24 and 48 h (Tables 3 and 4) and. It appears that recombinant Klotho suppresses the myocardial production of pro-inflammatory mediators via inhibition of NF-κB activation.

**Table 3:** Treatment with recombinant Klotho decreases myocardial cytokine and chemokine levels in aging endotoxemic mice

Cytokines	With Saline			With Klotho		
	Control	LPS 24 h	LPS 48 h	Control	LPS 24 h	LPS 48 h
MCP-1 (ng/mg)	110±11	864±148*	450±84*	106±23	491±42*^#^	268±69*^#^
KC (ng/mg)	94.5±7.1	1214±195*	743±104*	98.2±17	752±37*^#^	434±46*^#^
IL-6 (ng/mg)	81.8±4.7	468±52*	356±23*	78.8±10	375±23*^#^	260±31*^#^
IL-1β (pg/mg)	93.4±6.1	677±57*	417±35*	89.8±18	453±13*^#^	287±27*^#^
TNF-α (pg/mg)	20.1±2.7	439±69*	260±27*	22.3±6.4	260±21*^#^	157±17*^#^

Data are expressed as mean ± SE. n=6 in each of the 6 groups; *P<0.05 vs. control; ^#^*P*<0.05 vs. LPS+saline

**Table 4:** Treatment with recombinant Klotho decreases plasma cytokine and chemokine levels in aging endotoxemic mice

Cytokines	With Saline			With Klotho		
	Control	LPS 24 h	LPS 48 h	Control	LPS 24 h	LPS 48 h
MCP-1 (ng/ml)	111.2±19	1191±147*	442±202*	102±27	640±151*^#^	377±37.8*^#^
KC (ng/ml)	63.7±11	540±57*	288±36*	67.1±19	440±37*^#^	287±22*^#^
IL-6 (ng/ml)	35.4±6.6	3040±564*	993±99*	37.4±8.4	1955±212*^#^	315±67*^#^
IL-1β (pg/ml)	44.3±5.6	1116±105*	467±107*	42.8±7.9	561±81*^#^	375±73*^#^
TNF-α (pg/ml)	34.3±6.8	1099±109*	458±66*	35.4±7.2	739±73*^#^	261±65*^#^

Data are expressed as mean ± SE. n=6 in each of the 6 groups; *P<0.05 vs. control; ^#^*P*<0.05 vs. LPS+saline

### HSP70 suppresses macrophage NF-κB activation caused by LPS stimulation

**Figure 6:**
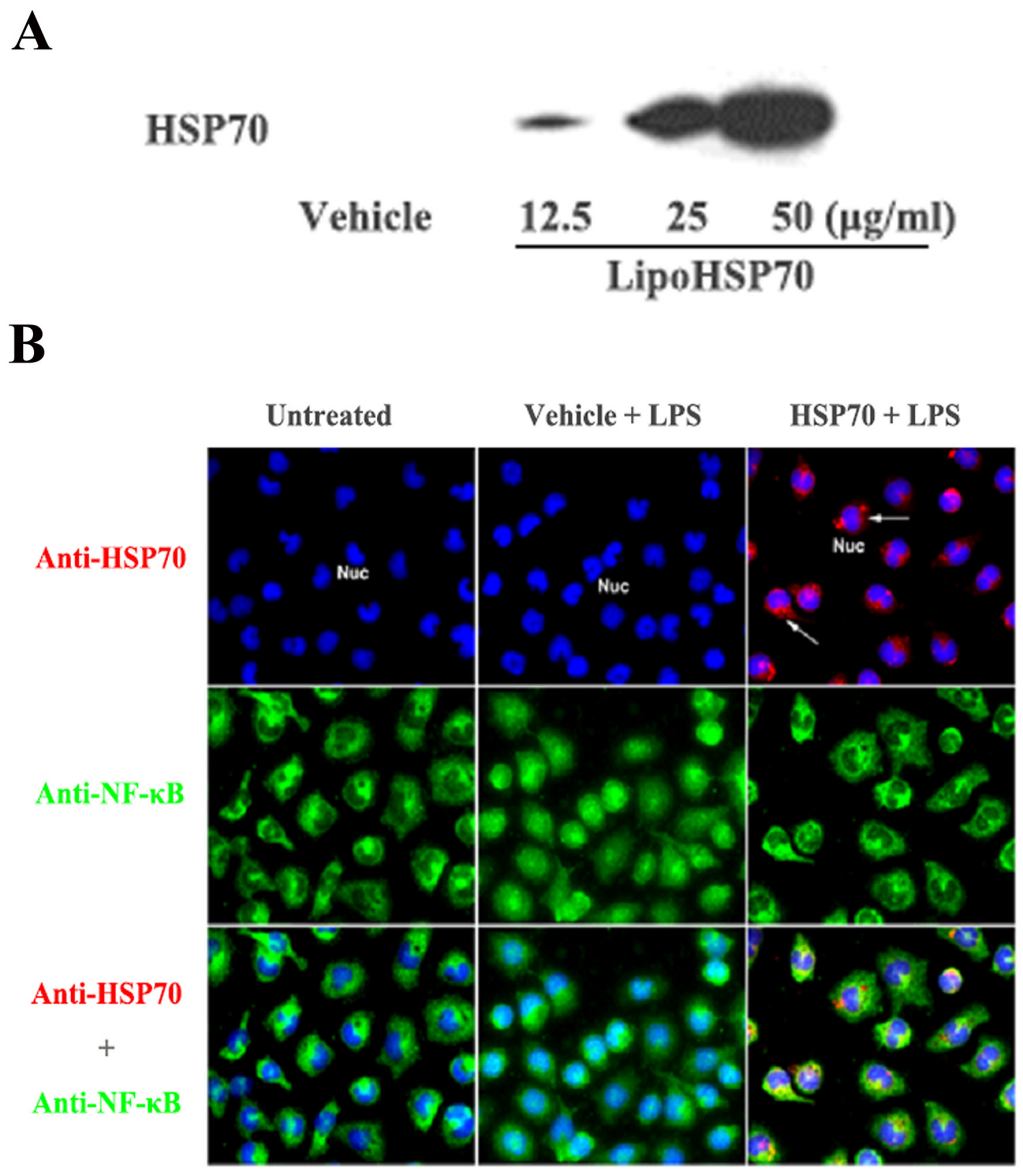
HSP70 suppresses LPS-induced NF-κB activation **A**. A representative immunoblot of 3 separate experiments shows that treatment with liposomal HSP70 dose-dependently increases cellular HSP70 levels in primary macrophages isolated from mouse peritoneal cavity. **B**. Representative immunofluorescence images of 3 separate experiments show that HSP70 (red, arrow) delivered into macrophages has a perinuclear distribution pattern and suppresses NF-κB (green) intranuclear translocation following LPS (0.2 μg/ml) stimulation. Objective 40x.

As the data presented above indicate a link between HSP70 and Klotho, we examined whether HSP70 modulates NF-κB activation, using liposomal delivery of recombinant HSP70 to primary macrophages that have low levels of HSP70 and high sensitivities to TLR4 stimulation. While HSP70 was barely detectable in macrophages treated with control liposomes (liposomes+saline), treatment with liposomal HSP70 increased cellular HSP70 levels in a dose-dependent fashion (Figure 6A). Liposomal HSP70 at 25 μg/ml markedly increased cellular HSP70 levels. Immunofluorescence staining confirmed increased HSP70 levels in cells treated with 25 μg/ml of liposomal HSP70 and revealed that the delivered HSP70 is primarily in the cytoplasm and the perinuclear regions (Figure 6B). Interestingly, NF-κB intranuclear translocation following LPS stimulation was essentially abrogated in cells with higher HSP70 levels (Figure 6B).

## DISCUSSION

Major surgery that has been performed more frequently on the elderly can cause sterile endotoxemia. Endotoxemia frequently results in cardiac dysfunction and has a significant impact on post-surgery outcomes [1, 24, 25]. Thus, understanding of the impact of endotoxemia on tissue inflammatory responses and organ function in aging subjects is helpful for improving clinical outcomes in the elderly undergoing major surgery.

Previous studies demonstrate that anti-aging protein Klotho has an anti-inflammatory effect in animal models of tissue ischemia and reperfusion injury [26] and aging is associated with relative Klotho deficiency [27]. It has been documented that the myocardial inflammatory responses to endotoxin are enhanced in old animals and such responses result in more severe cardiac dysfunction [2–5]. The current study focused on the role altered Klotho levels in aging-related myocardial inflammatory responses and cardiac dysfunction, and we are particularly interested in the effect of Klotho on endotoxemic pathology in aging subjects, as well as the therapeutic potential of recombinant Klotho in mitigation of aging-related endotoxemic cardiac dysfunction. The present study provided several novel findings. First, Klotho protein is present in the myocardial tissue, and baseline Klotho levels in aging hearts are lower compared to those of adult hearts. Second, endotoxemia causes a reduction of myocardial Klotho levels in both adult and aging hearts, and the impact of endotoxemia is greater in aging hearts. Third, myocardial Klotho levels fail to recover during the experimental period in aging hearts, resulting in extremely low myocardial Klotho levels accompanied by higher myocardial levels of pro-inflammatory cytokines and more severe LV functional injury. Lastly, treatment of aging endotoxemic mice with recombinant Klotho reduces cytokine levels and improves cardiac functional performance. It appears that aging-related relative Klotho deficiency and down-regulation of Klotho by endotoxemia, particularly in aging hearts, plays an important role in the mechanism of the augmented myocardial inflammatory responses and more severe cardiac dysfunction observed in aging endotoxemic mice. Recombinant Klotho may have therapeutic potential for modulation of myocardial inflammatory responses to endotoxemia and amelioration of endotoxemic cardiac dysfunction in aging subjects.

We previously reported that old mice had enhanced myocardial inflammatory responses to endotoxemia that result in exaggerated cardiac contractile depression at 6 h after administration of endotoxin [5]. In the present study, we extended the time trial to 24 and 48 h following administration of endotoxin. Cardiac function examined using a pressure-volume microcatheter is moderately decreased at 24 and 48 h in adult mice. In aging mice, however, endotoxemia causes more severe cardiac dysfunction at both time points. Cardiac functional injury in aging endotoxemic mice is accompanied by greater levels of cytokines and chemokines in the myocardium and plasma at 24 and 48 h. Similarly, aging hearts display higher and prolonged NF-κB activation in comparison to adult hearts. Thus, more severe cardiac dysfunction in aging mice is associated with greater and prolonged myocardial inflammatory responses. As pro-inflammatory cytokines are cardiodepressive [5, 8, 28], it is reasonable to postulate that greater levels of pro-inflammatory cytokines in the myocardium of aging mice are responsible, at least in part, for the more severe cardiac dysfunction.

The aging process is characterized by a chronic oxidation-inflammation state [29]. While multiple factors contribute to the aging process and/or accelerate aging, the role of Klotho in aging has attracted great attention [30, 31]. Recognized functions of Klotho include the regulation of energy metabolism, modulation of calcium homeostasis and anti-oxidative stress [14, 15, 32–34]. In addition, this anti-aging protein has been found to play a role in anti-inflammation [26]. Interestingly, we observed that Klotho protein is detectable in the heart. The levels of Klotho are lower in aging hearts than those in adult hearts. Thus, Klotho protein is also present in the heart and aging affects myocardial Klotho levels. A similar age-related difference in Klotho levels is observed in the kidney, but not in the liver. It is possible that liver-derived Klotho becomes an important source of extracellular Klotho in endotoxemic aging mice where Klotho levels in the kidneys and heart are significantly reduced.

We report here for the first time that endotoxemia decreases myocardial Klotho levels. While myocardial Klotho levels can recover to the baseline over time in adult endotoxemic mice, they fail to recover over the same time period in aging mice, which results in extremely low Klotho levels in aging hearts during endotoxemia. The lower myocardial Klotho levels are associated with greater NF-κB activation and augmented elevation of cytokine levels. It should be noted NF-κB activation is transient, but elevated cytokine levels last for a longer period. In this regard, NF-κB serves as an initiation signal; the production of pro-inflammatory mediators may not be shut off after NF-B is inactivated and cytokine protein degradation takes time. Thus, the elevation of cytokines is prolonged and pro-inflammatory mediators have relatively longer effects.

Treatment of aging endotoxemic mice with recombinant Klotho significantly reduces myocardial NF-κB activity and cytokine levels. These data support the notion of an interaction between Klotho and inflammation. Namely, inflammation can down-regulate Klotho levels and Klotho negatively modulates inflammation. In this regard, previous studies have shown that Klotho suppresses TNF-α-induced expression of adhesion molecules in endothelial cells [18] and pro-inflammatory cytokines reduce Klotho expression in the kidney [19, 20, 35]. It remains unclear from the present study how Klotho suppresses NF-κB activation and the inflammatory responses. Although extensive studies have yielded some information regarding signaling pathways activated by Klotho, no specific receptor for soluble Klotho has been identified up to date. However, it has been reported that soluble Klotho could modulate the PI3K/Akt and Wnt/β-catenin pathways [15] and these pathways are involved in cellular inflammatory responses to stimuli. Future studies are needed to elucidate the mechanism by which Klotho suppresses NF-κB-mediated inflammatory responses.

HSP70 is an inducible heat shock protein and protects cells and tissue against stress and injury [20, 21, 36]. HSP70 has also been indicated to be a biomarker of lifespan [37]. We observed that aging hearts have lower levels of HSP70 which is associated with lower Klotho levels. When myocardial Klotho levels in aging hearts are down-regulated by endotoxemia, the levels of myocardial HSP70 are also further reduced. Further, treatment of aging endotoxemic mice with recombinant Klotho prevents the reduction of myocardial HSP70 levels. All of these observations indicate that Klotho modulates HSP70 levels in the heart. Similarly, Sugiura and colleagues observed that over-expression of Klotho in adult mice increases HSP70 levels in the kidney [38]. As HSP70 is cardioprotective [39], it is likely that lower levels of HSP70 in aging endotoxemic mice contribute to the mechanism of cardiac functional injury and that preservation of myocardial HSP70 levels is one of the mechanisms underlying the cardioprotective effect of Klotho. However, the mechanism by which Klotho modulates HSP70 levels in the aging heart remains unclear from the present study. It is possible that Klotho is required to preserve myocardial HSP70 levels, particularly during endotoxemia. Alternatively, Klotho may up-regulate HSP70 expression in the heart. Further studies are needed to address the mechanism.

Nevertheless, intracellular HSP70 appears to negatively modulate pro-inflammatory signaling. In this regard, the data generated from the *in vitro* experiment show that liposomal delivery of recombinant HSP70 into cultured macrophages elevates cell-associated HSP70 levels and suppresses NF-κB activation in response to subsequent endotoxin stimulation. It is interesting that HSP70 is perinuclear. It is possible that HSP70 attenuates NF-κB intranuclear localization by preventing its translocation into the nucleus. Since recombinant Klotho can preserve myocardial HSP70 levels and suppress myocardial inflammatory responses characterized by NF-κB activation and inflammatory cytokine production, it is likely that preservation of HSP70 levels by Klotho is one of the mechanisms underlying its effects on inflammation and cardiac dysfunction in aging endotoxemic mice.

## CONCLUSIONS

Relative Klotho deficiency is, at least partly, responsible for the augmented inflammatory responses and more severe cardiac functional injury in aging endotoxemic mice. Post-treatment with recombinant Klotho preserves myocardial HSP70 levels, suppresses myocardial inflammatory responses and improves cardiac function in aging endotoxemic mice. The anti-inflammatory and cardioprotective mechanism of Klotho appears to involve HSP70. These novel findings suggest that Klotho has therapeutic potential for amelioration of endotoxemic cardiac dysfunction in aging subjects.

## MATERIALS AND METHODS

### Ethics statement

The experiments were approved by the Institutional Animal Care and Use Committee of the University of Colorado Denver, and this investigation conforms to the Guide for the Care and Use of Laboratory Animals (National Research Council, revised 1996).

### Chemicals and reagents

Antibodies against Klotho, HSP70 and β-actin were purchased from Santa Cruz Biotechnology, Inc (Dallas, TX). Antibodies against NF-κB p65 were purchased from Cell Signaling, Inc (Beverly, MA). Recombinant HSP70 (endotoxin-free) was purchased from Assay Design (Ann Arbor, MI). Enzyme-linked immunosorbent assay (ELISA) kits for MCP-1, KC, TNF-α, IL-1β and IL-6 were purchased from R&D System (Minneapolis, MN). Recombinant Klotho was purchased from Abcam Inc. (Cambridge, MA). TransAM NF-κB activity (p65 DNA-binding) assay kit was purchased from Active Motif (Carlsbad, CA). D-MEM/F-12 medium was purchased from Lonza (Walkersville, MD). Lipopolysaccharide (LPS, Escherichia coli 0111:B4) and all other chemicals and reagents were purchased from Sigma-Aldrich Chemical (St Louis, MO).

### Animals and treatment

Male adult (4 to 6 months) and aging (18 to 20 months) C57BL/6 mice were obtained from the Jackson Laboratory (Bar Harbor, Maine, USA) and National Institute on Aging (Bethesda, MD, USA), respectively. Mice were acclimated for 14 days in a 12:12 h light-dark cycle with free access to water and regular chow diet. The experiments were approved by the Institutional Animal Care and Use Committee of the University of Colorado Denver, and this investigation conforms to the Guide for the Care and Use of Laboratory Animals (National Research Council, revised 1996).

Adult and aging mice were assigned to saline group (control group) or LPS group (n = 8 in each time point). All treatments were performed in the morning. LPS (0.5 mg/kg) was injected through a tail vein. Control animals were treated with the same volume of sterile normal saline. Plasma and myocardial tissue were collected following left ventricle (LV) function analysis using a pressure-volume microcatheter at 24 and 48 h after administration of LPS or saline. No mortality was observed in adult endotoxemic mice or aging endotoxemic mice treated with recombinant Klotho, but the same endotoxemia protocol resulted in a 20% mortality in aging mice without treatment with recombinant Klotho.

Additional aging mice were assigned to LPS+saline group or LPS+Klotho group (n = 6 in each time point). Normal saline or recombinant Klotho (10 μg/kg, iv) was administered 30 min after administration of LPS (0.5 mg/kg, iv). Plasma and myocardial tissue were collected at 24 and 48 h after administration of LPS following LV function analysis.

### Measurement of cardiac function

Cardiac function was assessed as described previously [40, 41]. Briefly, mice were anesthetized with isoflurane inhalation (2% isoflurane mixed with pure oxygen), and anticoagulated with heparin (Elkins-Sinn, Cherry Hill, NJ; 1,000 units/kg, ip). Animals were laid supine on a heating blanket and core body temperature was maintained at 37 ± 0.5°C. A pressure-volume microcatheter (Millar Instruments, Houston, TX; 1 F) was inserted into the LV through the right common carotid artery. Pressure-volume loop was recorded using the MPVS-400 system with the aid of PVAN software (Millar Instruments, Houston, TX). Heart rate, LV developed pressure, ejection fraction and cardiac output were analyzed.

### Immunoblotting

Immunoblotting was applied to determine Klotho protein levels in the myocardium, kidney and liver Klotho, as well as HSP 70 protein levels in the myocardium. Proteins in tissue homogenate were separated on gradient (4%–20%) mini-gels and transferred onto nitrocellulose membranes (Bio-Rad Laboratories, Hercules, CA). The membranes were blocked with 5% skim milk solution for 1 hour at room temperature. The blocked membranes were incubated with a primary antibody against a protein of interest overnight in a 4°C cold room. After washing with phosphate-buffered saline (PBS) containing 0.05% Tween 20, the membranes were incubated with a peroxidase-linked secondary antibody specific to the primary antibody for 1 hour at room temperature. After further wash, membranes were treated with enhanced chemiluminescence reagents. The membranes were exposed on x-ray films. Image J (Wayne Rasband, National Institutes of Health, Bethesda, MD) was used to analyze band density.

### ELISA

ELISA kits were utilized to quantify monocyte chemoattractant protein-1 (MCP-1), keratinocyte chemoattractant (KC), TNF-α, interleukin (IL)-1β and IL-6 levels in plasma and myocardial tissue homogenate. Klotho levels in myocardial tissue homogenate were also analyzed using an ELISA kit. Samples and standards were prepared according to manufacturers’ instructions. Absorbance of standards and samples was determined spectrophotometrically at 450 nm, using a microplate reader (Bio-Rad Laboratories, Inc, Hercules, CA). Results were plotted against the linear portion of a standard curve.

### NF-κB activation assay

NF-κB activity (p65 DNA-binding) was measured using the TransAM NF-κB assay kit following the manufacturer's instruction. Absorbance of samples was determined spectrophotometrically at 450 nm, using a microplate reader (Bio-Rad Laboratories, Inc, Hercules, CA).

### Macrophage isolation and treatment

Since primary macrophages have low levels of HSP70 and display high sensitivity to the pro-inflammatory effect of LPS, we examined the effect of liposomal delivery of recombinant HSP70 on macrophage NF-κB activation.

Macrophages were collected from mouse peritoneal cavity by lavage as previously described [42]. Briefly, mice were anesthetized with 60 mg/kg of sodium pentobarbital intravenously. Their peritoneal cavities were flushed with cold (4°C) serum-free D-MEM/F-12 medium. Medium was recovered and cells were harvested by centrifugation at 1,100 rpm, 4°C for 10 minutes (IEC Centra MP4R, International Equipment Co., Needham Hts, MA). Cells were washed with serum-free medium and then suspended in full D-MEM/F-12 medium (containing 10% fetal bovine serum and 150 μg/ml of gentamycin). Cells were plated onto culture dishes or chamber slides and incubated in an incubator (5% CO2/95% atmosphere air) at 37°C for 2 hours. Non-adherent cells were removed by 3 washes with culture medium.

Recombinant HSP70 was delivered to cells using liposomes. A thin film of lipids (2.0 mg of egg L-α-phosphatidylcholine, 0.5 mg of cholesterol, 0.5 mg of dioleoyl 1, 2-diacyl-3-trimethyl ammonium-propane and 0.5 mg of dioleoylphosphatidylethanolamine) was prepared in a glass tube. HSP70 solution was added to the dried lipid in the tube, and a suspension of liposomal HSP70 is prepared by alternating sonication and vortex. Equal number of cells on 12-well plates were treated with liposomes containing recombinant HSP70 or liposomes containing normal saline (vehicle) for 2 hours followed by a 2-hour period of incubation in the medium without liposomes. The final concentration of HSP70 in the medium was 0, 12.5, 25 or 50 μg/ml. A batch of treated cells was collected for the assessment of cellular HSP70 levels by immunoblotting. Since no difference in cell density among treatments was observed following this short period of treatment, the levels of HSP70 were compared among treatments based on equal cell density. Other batches of cells were stimulated with LPS (0.2 μg/ml) for 2 h for examining NFκB intranuclear translocation.

### Immunofluorescence staining

Dual immunofluorescence staining was performed, as described previously [43], to localize HSP70 and NF-κB p65 in cultured macrophages. After permeabilization of cell membranes with an ethanol/acetone mixture, cells on chamber slides were fixed in 4% paraformaldehyde, incubated overnight at 4°C with a rat monoclonal antibody against HSP70 and a rabbit polyclonal antibody against NF-κB p65. After washing with PBS, cells were incubated with Cy3-tagged goat antibody against rat IgG (labeling HSP70 red) and 488-tagged goat antibody against rabbit IgG (labeling NF-κB p65 green). Nuclei were stained with bis-benzimide (blue). Microscopy was performed with a Leica DM 5500 digital microscope (Leica Microscopy und System GmbH, Wechsler, Germany).

### Statistical analysis

Data are presented as mean ± standard error (SE). Statistical analysis was performed using StatView software (Abacus Concepts, Calabasas, CA, USA). Analysis of variance (ANOVA) with Fisher post-hoc test was used to analyze differences between experimental groups, and differences were confirmed using the Mann-Whitney U-test. Statistical significance was defined as *P*≤0.05.

## KEY MESSAGES

1. Aging hearts have lower Klotho levels, and endotoxemia further reduces myocardial Klotho levels. Treatment with recombinant Klotho suppresses myocardial inflammatory responses and improves cardiac function in aging endotoxemic mice.

2. Relative Klotho deficiency in the myocardium of aging endotoxemic mice is associated with down-regulation of HSP70 in the heart. Treatment with Klotho preserves myocardial HSP70 levels.

3. HSP70 is involved in suppression of the inflammatory response to endotoxin.

## Abbreviations

TLR4: Toll like receptor 4
TNF-α: tumor necrosis factor-α
IL: interleukin
HSP70: heat shock protein 70
LPS: lipopolysaccharide
PBS: phosphate-buffer saline
MCP-1: chemoattractant protein-1
KC: keratinocyte chemoattractant
NF-κB: nuclear factor-κB

## References

[1] Charbonney E, Tsang JY, Li Y, Klein D, Duque P, Romaschin A, Marshall JC (2016). Endotoxemia Following Multiple Trauma: Risk Factors and Prognostic Implications. Crit Care Med.

[2] Saito H, Papaconstantinou J (2001). Age-associated differences in cardiovascular inflammatory gene induction during endotoxic stress. J Biol Chem.

[3] Starr ME, Ueda J, Takahashi H, Weiler H, Esmon CT, Evers BM, Saito H (2010). Age-dependent vulnerability to endotoxemia is associated with reduction of anticoagulant factors activated protein C and thrombomodulin. Blood.

[4] Leong J, Zhou M, Jacob A, Wang P (2010). Aging-related hyperinflammation in endotoxemia is mediated by the alpha2A-adrenoceptor and CD14/TLR4 pathways. Life Sci.

[5] Slimani H, Zhai Y, Yousif NG, Ao L, Zeng Q, Fullerton DA, Meng X (2014). Enhanced monocyte chemoattractant protein-1 production in aging mice exaggerates cardiac depression during endotoxemia. Crit Care.

[6] Akira S, Takeda K, Kaisho T (2001). Toll-like receptors: critical proteins linking innate and acquired immunity. Nat Immunol.

[7] Weisensee D, Bereiter-Hahn J, Schoeppe W, Low-Friedrich I (1993). Effects of cytokines on the contractility of cultured cardiac myocytes. Int J Immunopharmacol.

[8] Meng X, Ao L, Meldrum DR, Cain BS, Shames BD, Selzman CH, Banerjee A, Harken AH (1998). TNF-alpha and myocardial depression in endotoxemic rats: temporal discordance of an obligatory relationship. Am J Physiol.

[9] Kumar A, Haery C, Parrillo JE (2000). Myocardial dysfunction in septic shock. Crit Care Clin.

[10] Ao L, Song Y, Fullerton DA, Dinarello CA, Meng X (2007). The interaction between myocardial depressant factors in endotoxemic cardiac dysfunction: role of TNF-alpha in TLR4-mediated ICAM-1 expression. Cytokine.

[11] Kuro-o M, Matsumura Y, Aizawa H, Kawaguchi H, Suga T, Utsugi T, Ohyama Y, Kurabayashi M, Kaname T, Kume E, lwasake H, lida A, Shiraki-lida T (1997). Mutation of the mouse klotho gene leads to a syndrome resembling ageing. Nature.

[12] Lim K, Lu TS, Molostvov G, Lee C, Lam FT, Zehnder D, Hsiao LL (2012). Vascular Klotho deficiency potentiates the development of human artery calcification and mediates resistance to fibroblast growth factor 23. Circulation.

[13] Hu MC, Kuro-o M, Moe OW (2013). Renal and extrarenal actions of Klotho. Semin Nephrol.

[14] Imura A, Tsuji Y, Murata M, Maeda R, Kubota K, Iwano A, Obuse C, Togashi K, Tominaga M, Kita N, Tomiyama K, lijima J, Nabeshima Y (2007). alpha-Klotho as a regulator of calcium homeostasis. Science.

[15] Xu Y, Sun Z (2015). Molecular basis of Klotho: from gene to function in aging. Endocr Rev.

[16] Martin-Nunez E, Donate-Correa J, Muros-de-Fuentes M, Mora-Fernandez C, Navarro-Gonzalez JF (2014). Implications of Klotho in vascular health and disease. World J Cardiol.

[17] Xie J, Cha SK, An SW, Kuro OM, Birnbaumer L, Huang CL (2012). Cardioprotection by Klotho through downregulation of TRPC6 channels in the mouse heart. Nat Commun.

[18] Maekawa Y, Ishikawa K, Yasuda O, Oguro R, Hanasaki H, Kida I, Takemura Y, Ohishi M, Katsuya T, Rakugi H (2009). Klotho suppresses TNF-alpha-induced expression of adhesion molecules in the endothelium and attenuates NF-kappaB activation. Endocrine.

[19] Moreno JA, Izquierdo MC, Sanchez-Nino MD, Suarez-Alvarez B, Lopez-Larrea C, Jakubowski A, Blanco J, Ramirez R, Selgas R, Ruiz-Ortega M, Ruiz-Ortega M, Egido J, Ortiz A, Sanz AB (2011). The inflammatory cytokines TWEAK and TNFalpha reduce renal klotho expression through NFkappaB. J Am Soc Nephrol.

[20] ZhiQing Z, XinXing W, Jingbo G, Rui Z, Xiujie G, Yun Z, Lei W, Xue L, LingJia Q (2014). Effects of HIP in protection of HSP70 for stress-induced cardiomyocytes injury and its glucorticoid receptor pathway. Cell Stress Chaperones.

[21] Van Molle W, Wielockx B, Mahieu T, Takada M, Taniguchi T, Sekikawa K, Libert C (2002). HSP70 protects against TNF-induced lethal inflammatory shock. Immunity.

[22] Calderwood SK, Murshid A, Prince T (2009). The shock of aging: molecular chaperones and the heat shock response in longevity and aging--a mini-review. Gerontology.

[23] Singh R, Kolvraa S, Bross P, Christensen K, Gregersen N, Tan Q, Jensen UB, Eiberg H, Rattan SI (2006). Heat-shock protein 70 genes and human longevity: a view from Denmark. Ann N Y Acad Sci.

[24] Kritselis I, Tzanetakou V, Adamis G, Anthopoulos G, Antoniadou E, Bristianou M, Kotanidou A, Lignos M, Polyzos K, Retsas T, Sassopoulou P, Papaioannou AI, Sinapidis D (2013). The level of endotoxemia in sepsis varies in relation to the underlying infection: Impact on final outcome. Immunol Lett.

[25] Klein DJ, Briet F, Nisenbaum R, Romaschin AD, Mazer CD (2011). Endotoxemia related to cardiopulmonary bypass is associated with increased risk of infection after cardiac surgery: a prospective observational study. Crit Care.

[26] Hu MC, Shi M, Zhang J, Quinones H, Kuro-o M, Moe OW (2010). Klotho deficiency is an early biomarker of renal ischemia-reperfusion injury and its replacement is protective. Kidney Int.

[27] Manya H, Akasaka-Manya K, Endo T (2010). Klotho protein deficiency and aging. Geriatr Gerontol Int.

[28] Cha J, Wang Z, Ao L, Zou N, Dinarello CA, Banerjee A, Fullerton DA, Meng X (2008). Cytokines link Toll-like receptor 4 signaling to cardiac dysfunction after global myocardial ischemia. Ann Thorac Surg.

[29] M De la Fuente, Miquel J (2009). An update of the oxidation-inflammation theory of aging: the involvement of the immune system in oxi-inflamm-aging. Curr Pharm Des.

[30] Kuro-o M (2011). Klotho and the aging process. Korean J Intern Med.

[31] Barnes PJ (2015). Mechanisms of development of multimorbidity in the elderly. Eur Respir J.

[32] Razzaque MS (2012). The role of Klotho in energy metabolism. Nat Rev Endocrinol.

[33] Percy CJ, Power D, Gobe GC (2008). Renal ageing: changes in the cellular mechanism of energy metabolism and oxidant handling. Nephrology (Carlton).

[34] Ravikumar P, Ye J, Zhang J, Pinch SN, Hu MC, Kuro-o M, Hsia CC, Moe OW (2014). alpha-Klotho protects against oxidative damage in pulmonary epithelia. Am J Physiol Lung Cell Mol Physiol.

[35] Ohyama Y, Kurabayashi M, Masuda H, Nakamura T, Aihara Y, Kaname T, Suga T, Arai M, Aizawa H, Matsumura Y, Kuro-o M, Yi Nabeshima, Nagail R (1998). Molecular cloning of rat klotho cDNA: markedly decreased expression of klotho by acute inflammatory stress. Biochem Biophys Res Commun.

[36] Pasqua T, Filice E, Mazza R, Quintieri AM, M Carmela Cerra, Iannacone R, Melfi D, Indiveri C, Gattuso A, Angelone T (2015). Cardiac and hepatic role of r-AtHSP70: basal effects and protection against ischemic and sepsis conditions. J Cell Mol Med.

[37] I Martinez de Toda, De la Fuente M (2015). The role of Hsp70 in oxi-inflamm-aging and its use as a potential biomarker of lifespan. Biogerontology.

[38] Sugiura H, Yoshida T, Mitobe M, Yoshida S, Shiohira S, Nitta K, Tsuchiya K (2010). Klotho reduces apoptosis in experimental ischaemic acute kidney injury via HSP-70. Nephrol Dial Transplant.

[39] Meng X, Harken AH (2002). The interaction between Hsp70 and TNF-alpha expression: a novel mechanism for protection of the myocardium against post-injury depression. Shock.

[40] Pacher P, Nagayama T, Mukhopadhyay P, Batkai S, Kass DA (2008). Measurement of cardiac function using pressure-volume conductance catheter technique in mice and rats. Nat Protoc.

[41] Su X, Sykes JB, Ao L, Raeburn CD, Fullerton DA, Meng X (2010). Extracellular heat shock cognate protein 70 induces cardiac functional tolerance to endotoxin: differential effect on TNF-alpha and ICAM-1 levels in heart tissue. Cytokine.

[42] Heimbach JK, Reznikov LL, Calkins CM, Robinson TN, Dinarello CA, Harken AH, Meng X (2001). TNF receptor I is required for induction of macrophage heat shock protein 70. Am J Physiol Cell Physiol.

[43] Zeng Q, Jin C, Ao L, Cleveland JC, Song R, Xu D, Fullerton DA, Meng X (2012). Cross-talk between the Toll-like receptor 4 and Notch1 pathways augments the inflammatory response in the interstitial cells of stenotic human aortic valves. Circulation.

